# Population genetics of self-incompatibility in a clade of relict cliff-dwelling plant species

**DOI:** 10.1093/aobpla/plw029

**Published:** 2016-07-11

**Authors:** Jose L. Silva, Adrian C. Brennan, José A. Mejías

**Affiliations:** ^1^Departamento De Biología Vegetal Y Ecología, Universidad De Sevilla, Sevilla, CP 41012, España; ^2^School of Biological and Biomedical Sciences, University of Durham, Durham, UK

**Keywords:** Breakdown of SI, diallel crosses, index of self-incompatibility, *S* allele diversity, Sonchus section Pustulati, sporophytic self-incompatibility, resilience

## Abstract

The mating systems of species in small or fragmented populations impact upon their persistence. Small self-incompatible (SI) populations risk losing *S* allele diversity, responsible for the SI response, by drift thereby limiting mate availability and leading to population decline or SI system breakdown. But populations of relict and/or endemic species have resisted these demographic conditions over long periods suggesting their mating systems have adapted. To address a lack of empirical data on this topic, we studied the SI systems of three relict cliff-dwelling species of *Sonchus* section *Pustulati* (Asteraceae): *S. masguindalii*, *S. fragilis* and *S. pustulatus* in the western Mediterranean region. We performed controlled pollinations within and between individuals to measure index of SI (ISI) expression and identify *S* alleles in multiple population samples. *Sonchus masguindalii* and *S. pustulatus* showed strong SI (ISI = 0.6–1.0) compared to *S. fragilis* (ISI = 0.1–0.7). Just five *S* alleles were estimated for Spanish *S. pustulatus* and a moderate 11-15 *S* alleles for Moroccan *S. pustulatus* and *S. fragilis*, respectively. The fact that autonomous fruit set was generally improved by active self-pollination in self-compatible *S. fragilis* suggests that individuals with weak SI can show a wide range of outcrossing levels dependent on the degree of self or outcross pollen that pollinators bear. We conclude that frequent *S* allele dominance interactions that mask the incompatibility interactions of recessive *S* alleles leading to higher mate availability and partial breakdown of SI leading to mixed mating, both contribute to reproductive resilience in this group.

## Abstract (precis)

This study highlights the value of performing detailed mating system studies in plant species of high conservation value, such as the rare and relict species of *Sonchus* section *Pustulati* described here. This study adds to the evidence that outcrossing mating systems based on SSI are highly resilient even under long-term conditions of small, fragmented, and isolated populations, possibly due to mating system flexibility with the presence of some selfing and the fact that high cross-compatibility is achieved for relatively modest dominantly expressed S allele polymorphism. We highlight the importance of taking mating system factors into account as part of conservation efforts.

## Introduction

Self-incompatibility (SI) is a genetic barrier to inbreeding that is broadly distributed among hermaphroditic angiosperms (Busch and Schoen 2008). Two major types of self-incompatibility are recognized based on the genetic control of the incompatibility reaction: gametophytic (GSI) and sporophytic (SSI) systems. In both SI types, the incompatibility reactions are controlled by a linked cluster of genes collectively known as the ‘*S* locus’ (‘S’ denotes self-sterility), and individual plants that share alleles at this locus do not produce offspring in cross-pollinations (Richards 1997). In GSI, the more common type of SI, the incompatibility *S* allele phenotype is governed by the genotype of each single haploid pollen grain. In SSI, which has so far been detected in seven dicotyledonous families (Asteraceae, Betulaceae, Brassicaceae, Caryophyllaceae, Convolvulaceae, Polemoniaceae and Sterculiaceae; Hiscock and Kües 1999), the pollen incompatibility phenotype is genetically controlled by the plant that produces the pollen grains (i.e. the sporophyte) through the diploid pollen grain coat. In both cases, if the pollen of the donor plant is recognized by the receptor plant as own pollen (i.e. they share at least one allele, codominantly or dominantly expressed in the case of SSI), the receptor triggers the SI reaction. The molecular basis of SSI is best understood in Brassicaceae, where the maternal receptor and paternal ligand are known, and while the molecular basis of SSI in other families including Asteraceae seems to be distinct, a similar receptor-ligand ‘lock-and-key’ model of SSI is generally assumed (Allen *et al.* 2011).

Regardless of the type of SI, reproductive success in populations of SI species depends on the number and frequency of alleles at the *S* locus (Busch and Schoen 2008) and, in turn, the number of *S* alleles depends on the long-term population size and the mutation rate of the *S* locus (Wright 1939; Busch *et al.* 2014). *S* allele diversity may therefore have important consequences for population biology, and in large populations many *S* alleles are maintained by negative-frequency-dependent selection (Wright 1939; Schierup *et al.* 1997; Lawrence 2000). Meanwhile, in small or highly fragmented populations *S* allele diversity can be lost due to genetic drift (Wagenius *et al.* 2007). In this case, SI species can suffer from limited reproduction through scarcity of compatible mates (*S* Allee effect) or strong inbreeding depression following breakdown of SI (Byers and Meagher 1992; Willi and Fisher 2005; Glémin *et al.* 2005; Wagenius *et al.* 2007; Caujapé-Castell *et al.* 2008; Young and Pickup 2010; Leducq *et al.* 2010), which can lead to the extinction of rare or endemic taxa (Demauro 1993; Reinartz and Les 1994; Caujapé-Castells *et al.* 2008). The demographic consequences of the *S* Allee effect in SSI systems may be especially important in populations with fewer than 10 *S* alleles (Busch *et al.* 2014), whose spatial distribution within the population can also influence local reproductive success. These reproductive problems may be aggravated for insect pollinated species due to associated declines in specialist pollinators or limited pollinator attractiveness when rare (Rymer *et al.* 2005).

Recovery of reproductive assurance in SI plants subjected to strong mate limitation in declining or new populations is usually expected to involve selection for self-compatibility (SC) (Reinartz and Les 1994; Ortiz *et al.* 2006; Wagenius *et al.* 2007) that can be detected from low index of incompatibility (ISI) measures (Lloyd 1965). Nevertheless, selection for SC does not always occur as has been shown in cases of maintenance of SI through recent colonization events (Carr and Powel 1986; Kim *et al.* 1999; Brennan *et al.* 2002, 2003; Miller *et al.* 2008). In some cases, alternative solutions to increasing mate availability have been found such as increasing dominance interactions among remaining *S* alleles to increase cross-compatibility in SSI systems (Brennan *et al.* 2002). Increased *S* allele dominance leads to increased cross-compatibility because more recessively expressed *S* alleles are masked, resulting in more compatible crosses between individuals that share these recessive *S* alleles. Alternatively, SC alone might not be sufficient to increase self-fertilization, if the plant species still requires pollinators for effective pollen transfer from anthers to stigmas (Gandhi *et al.* 2005). Moreover, between the two ends of the mating system spectrum (SI and SC), sexual systems of intermediate nature that seem to combine the advantages of both selfing and outcrossing, i.e. the partial self-incompatibility (pSI) systems, have frequently been reported (Levin 1996; Igic and Busch 2013).

Studies of populations undergoing stressful conditions due to major demographic changes (fragmentation, decline, founder events or colonization) have been invaluable to our understanding of the factors shaping mating system evolution (Willi and Fisher 2005; Nielsen *et al.* 2007; Wagenius *et al.* 2007; Busch *et al.* 2010; Young and Pickup 2010; Leducq *et al.* 2010). However, not all species with small and/or fragmented populations are in the process of major recent demographic upheaval (Mable and Adam 2007; Hoebe *et al.* 2009). Many endemic species with narrow environmental requirements are relicts, i.e. have stably persisted for long periods maintaining small and fragmented distribution ranges which often reflect relatively old vicariance events. This makes them potentially very interesting models to understand mating system evolution in small isolated populations over long sustained periods. In addition, these species also make important contributions to the biodiversity of many regions and are increasingly of conservation concern due to habitat loss (Thompson 2005). Mating system evolution and particularly the maintenance of SI systems have hardly been studied in these species to date.

**Figure 1.**
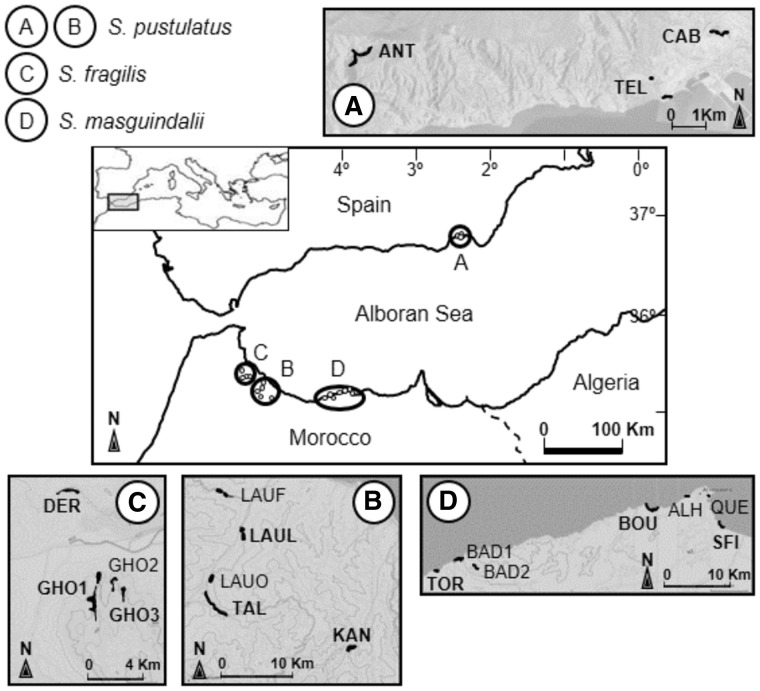
Distribution range of the species of *Sonchus* section *Pustulati*, locations of all known populations, and area of occupancy (Silva *et al.* 2015b). Sampled populations are in bold.

*Sonchus pustulatus*, *S. fragilis* and *S. masguindalii* constitute the well-supported clade *Sonchus* section *Pustulati* (Asteraceae, Cichorieae; Boulos 1973; Kim *et al.* 2007; Silva *et al.* 2015a). These species are narrow endemics restricted to both sides of the western Mediterranean Basin (Spain and Morocco) occurring on localized cliffs at low altitude (Fig. 1; Silva *et al.* 2015*b*). All three species are found in North Africa, where they are considered to be very rare (Fennane and Ibn Tattou 1998). *S. pustulatus* also occurs in the SE Iberian Peninsula, where it is categorized as ‘critically endangered’ (Cueto *et al.* 2003; Silva *et al.* 2015*b*). Phylogenetic and phylogeographic data suggest that these taxa are relicts of the late Tertiary (Silva *et al.* 2015*a*). Their restricted and disjunct distribution seems to be related to old geological events of large biogeographic impact: the Messinian Salinity Crisis and the subsequent Zanclean reflooding of the Mediterranean Basin 5.96–3.60 million years ago (Krijgsman *et al.* 1999; Rosenbaum *et al.* 2002; Fauquette *et al.* 2006), and the subsequent establishment of the Mediterranean climate (3.2–2.8 Ma; Suc, 1984). These relict and endemic taxa seem to have suffered from erosion of genetic diversity by genetic drift during long periods of small population size that is reflected in previous observations of relatively low genetic diversity (*H*_E _=_ _0.088–0.177; Silva *et al.* 2015*a*). Therefore, it is also of interest to investigate their SI mating systems. These species are pollinated by generalist insects (Silva 2014) and preliminary hand pollinations suggested that *S. pustulatus* in Spain is SI (Mejías 1992). We predict that strong long-term genetic drift could have led to limited *S* allele diversity, capacity of selfing or frequent dominance interactions among remaining *S* alleles.

Here, we report a detailed population genetic study of the mating system in the species of *Sonchus* section *Pustulati* based on an intensive program of hand pollinations. Though in recent decades, the development of biochemical and molecular techniques for SSI in *Brassica* (Gaude *et al.* 1991; Brace *et al.* 1993) has allowed a faster and less tedious *S* allele identification than using classical diallel crosses (Glémin *et al.* 2005), the molecular mechanism of SSI in the Brassicaceae is not shared by the Asteraceae (Allen *et al.* 2011; Gounthier *et al.* 2013). Fortunately, a recent study with *Senecio* species showed how extensive controlled crossing surveys can be applied to investigate the SSI systems in species of particular ecological or evolutionary interest (Brennan *et al.* 2013). Based on this experimental approach the main goals of this study were to investigate across multiple relict *Sonchus* populations and species: (i) variation in the strength of SSI within and among populations of these relict species, (ii) dependency on insects for successful pollination, (iii) *S* allele diversity and (iv) *S* allele dominance interactions. We interpret and discuss our results in terms of mating system responses to the past and present demographic conditions faced by these species.

## Methods

### Plant species

The species *Sonchus pustulatus*, *S. fragilis* and *S. masguindalii* constitute the section *Pustulati* of the subgenus *Sonchus* and are all diploid (*n* = 9, 2*n* = 18; Mejías and Andrés 2004; Vogt and Oberprieler 2008). They are perennial suffrutescent chamaephytic cliff plants, usually procumbent, which develop moderate-long branches, particularly lignified in *S. masguindalii* (Silva *et al.* 2015*b*). Depending on the species, individuals range approximately from only 5 to 200 cm^2^ in surface and can reach up to 45 cm in height, with intricate branching. They commonly attain sexual maturity during the first year of life (Silva *et al.* 2015*b*) and mainly flower in spring. Flower heads are terminal, solitary or in groups from two to four, with bright yellow ligule florets. All florets are hermaphrodites and protandrous, which develop centripetally over a period of 3–6 days. Flower heads comprise 30–120 florets in the case of *S. pustulatus* and *S. fragilis*, and 60–250 florets in *S. masguindalii*. Fruits are achenes with a short-lasting pappus, released during late spring and early summer. Currently, there are only 19 known populations of these species, which are located in four small non-overlapped distribution areas restricted to the Baetic-Rifan geological complex in the western Mediterranean Basin (Silva *et al.* 2015*b*).

### Plant sampling and pollinations

In May and June 2008, we collected and georeferenced 281 plants (128 as cuttings and 153 as flower heads with mature seeds) from 12 populations (Fig. 1 and Table 1). We collected at a minimum distance of 20 m between plants to avoid collecting siblings and up to a maximum of 100 m apart in order to obtain a spatially representative sample of each population.

**Table 1.** General reproductive behaviour of plants from representative populations of the species of *Sonchus* section *Pustulati*. Fruit (achene) set after pollinations: *ASP* (autonomous self-pollination), *FSP* (hand forced self-pollination) and *MCP* (hand multiple individual cross-pollination). ISI, index of self-incompatibility (Lloyd 1965; Raduski et al. 2011). ISI states of taxa and populations were addressed according to the same criteria as for individuals (see ‘Methods’ section): SC (self-compatibility, mean ISI < 0.2), pSI (partial self-incompatibility, 0.2 ≥ mean ISI > 0.8) and SI (self-incompatibility, mean ISI ≥ 0.8). SE, standard error; N, sampled individuals.

Taxa and populations	Mean fruit set ± SE (*N*)			Mean ISI ± SE (*N*)	ISI states	Origin
	*ASP*	*FSP*	*MCP*			
*S. pustulatus* SE Spain	*0.04 ± 0.014 (66)*	*0.09 ± 0.024 (61)*	*0.46 ± 0.026 (58)*	*0.84 ± 0.11 (56)*	*SI*	
ANT	0.00 ± 0.001 (15)	0.07 ± 0.014 (14)	0.54 ± 0.025 (15)	0.87 ± 0.032 (14)	SI	Cuttings
TEL	0.06 ± 0.024 (14)	0.14 ± 0.056 (14)	0.33 ± 0.046 (12)	0.60 ± 0.161 (12)	pSI	Cuttings
CAB	0.04 ± 0.023 (37)	0.08 ± 0.037 (33)	0.47 ± 0.039 (31)	0.84 ± 0.001 (30)	SI	Seeds & Cuttings
*S. pustulatus* W Rif, Morocco	*0.00 ± 0.000 (68)*	*0.04 ± 0.016 (66)*	*0.61 ± 0.021 (55)*	*0.90 ± 0.12 (53)*	*SI*	
LAUL	0.00 ± 0.001 (20)	0.08 ± 0.031 (20)	0.68 ± 0.030 (20)	0.87 ± 0.051 (20)	SI	Seeds
TAL	0.00 ± 0.001 (34)	0.01 ± 0.007 (34)	0.55 ± 0.028 (22)	0.96 ± 0.021 (21)	SI	Seeds & Cuttings
KAN	0.00 ± 0.000 (14)	0.11 ± 0.067 (12)	0.59 ± 0.043 (13)	0.84 ± 0.096 (12)	SI	Cuttings
*S. fragilis* NW Rif, Morocco	*0.20 ± 0.026 (84)*	*0.43 ± 0.037 (84)*	*0.73 ± 0.017 (75)*	*0.42 ± 0.05 (75)*	*pSI*	
GHO1	0.13 ± 0.030 (44)	0.27 ± 0.049 (44)	0.71 ± 0.024 (35)	0.67 ± 0.078 (35)	pSI	Cuttings
GHO3	0.17 ± 0.036 (20)	0.48 ± 0.057 (20)	0.69 ± 0.047 (20)	0.29 ± 0.079 (20)	pSI	Seeds
DER	0.42 ± 0.059 (20)	0.72 ± 0.055 (20)	0.82 ± 0.046 (20)	0.11 ± 0.059 (20)	SC	Seeds
*S. masguindalii* Central Rif, Morocco	*0.02 ± 0.012 (61)*	*0.05 ± 0.022 (58)*	*0.61 ± 0.033 (45)*	*0.84 ± 0.13 (43)*	*SI*	
TOR	0.00 ±0.000 (24)	0.00 ± 0.000 (23)	0.65 ± 0.041 (22)	1.00 ± 0.000 (21)	SI	Seeds
BOU	0.00 ± 0.001 (11)	0.02 ± 0.012 (8)	0.41 ± 0.106 (6)	0.96 ± 0.030 (6)	SI	Cuttings
SFI	0.05 ± 0.028 (26)	0.12 ± 0.044 (27)	0.62 ± 0.055 (17)	0.58 ± 0.157 (16)	pSI	Seeds

The following procedures and experiments were performed in the greenhouses of the General Services of the University of Seville, under standard light and warm conditions (14 h of light, 18–22°C). Plants were grown in plastic 9 cm diameter pots, with a substrate of peat and perlite (3:1 v/v) plus a solid organic fertilizer of slow release (Osmocote 12 months; 3.5 g/L of substratum) until they reached the reproductive stage (approx. 2–4 months). Then, the plants of each population were covered with canopies of 1.5 m of height made with a tulle mesh with 1.5 mm diameter pore with pheromone traps (yellow Atrapaxon plates) within the boxes to exclude and eliminate pollinators. We maintained all plants at similar sizes by manual pruning to avoid possible maternal effects in the seed production level.

We performed the following pollination treatments: *autonomous self-pollination* (*ASP*), in which the flower head was neither hand pollinated nor used as pollen donor; *hand forced self-pollination* (*FSP*), in which the flower head had one or several flower heads of the same individual as pollen donors; *hand multiple individual cross-pollination* (*MCP*), in which the flower head had several individuals from the same population as pollen donors; and *hand single individual cross-pollination* (*SCP*), in which the flower head had exclusively one individual from the same population as pollen donor. Pollinations were manually carried out with small cotton sticks (Ortiz *et al.* 2006) or when the flower heads could approach each other without breaking them, this method was substituted by gently brushing flowering heads together (Brennan *et al.* 2002). The procedure was repeated 3–4 times in each flower head during the whole anthesis period to ensure pollination of all florets. After the anthesis, the treated flower heads were individually covered with new clean tea-bags. These bags were then collected when flower heads had fully dehisced, approximately 1 month after flowering.

Fruit set was chosen as a reliable measure of the incompatibility response since incompatible pollinations usually resulted in little or no fruit set and between 0.3 and 1.0 of fruit set for a compatible cross (Hiscock 2000; Brennan *et al.* 2002; Ortiz *et al.* 2006). Fruit set (seed set in Asteraceae) was estimated according to the formula:

$$Fruit\;set=\frac{No.\;fertile\;fruits}{No.\;fertile\;fruits+No.\;sterile\;fruits}.$$

Fertile fruits appeared fatter and more pigmented than fruits containing an unfertilized ovule, which were thin and whitish-pale in colour [see **Supporting Information – Figure S1**]. We calculated the average fruit-set of the flower heads with the same pollination treatment for each individual.

### Strength of self-incompatibility

We independently applied the treatments *ASP*, *FSP* and *MCP* for the study of the strength of the SI over three to six flower heads per individual in each of the 12 populations of the *Sonchus* section *Pustulati* sampled. To this end, we used a total of 281 plants obtained both from seeds and cuttings (11–44 individuals per population; mean ± SE = 23.4 ± 2.99; Table 1). Approximately 1900 flower heads received the treatments *ASP*, *FSP* or *MCP* (including repeats) among these sampled individuals.

To assess the strength and variation of SI, we calculated, for each individual, the most widely reported quantitative measure of SI, the index of self-incompatibility (ISI; Lloyd 1965; Raduski *et al.* 2011):

ISI=1- Relative selfed success/Relative outcrossed success,

where relative selfed or outcrossed success is defined as the fruit set by means of *FSP* and *MCP* treatments, respectively. We had previously observed under the binocular magnifier that spontaneous self-pollination can occur under the *ASP* treatment by means of the nystinastic movements of the flower heads (daily opening and closing) during anthesis. However, the *FSP* treatment ensures that the pollen is deposited on the stigmatic papilla where the SSI response occurs. Historically, species with ISI values above 0.8 have been classified as SI (Bawa 1974). We classified the breeding system of individuals into three states according to their ISI values, following Raduski *et al.* (2011): self-incompatibility (SI; ISI ≥ 0.8), partial self-incompatibility (pSI; 0.2 < ISI < 0.8) and self-compatibility (SC; ISI ≤ 0.2) and calculated the proportion of SC and pSI plants in each of the 12 populations. 

### Identification of incompatibility groups and *S* allele diversity in natural populations

The *SCP* treatment was applied to estimate the *S* allele diversity in three independent diallels in the Spanish population ANT of *S. pustulatus*, the Moroccan population TAL of *S. pustulatus* and the population GHO1 of *S. fragilis*. To this end, we used 14, 14 and 11 individuals in each diallel, respectively, all obtained from cuttings.

We only chose highly self-incompatible individuals (ISI ≥ 0.8) because the *SCP* treatment could not avoid self-pollination. Each *SCP* cross was reciprocally repeated between individual pairs from two to six times until three independent full cross diallels had been achieved. Approximately 1200 flower heads received the *SCP* treatment, including repeats.

Compatibility phenotypes were scored according to fruit set after *SCP* crosses. We considered a cross between two individuals to be incompatible (−) when the fruit set after the *SCP* treatment was less than 0.10; indeterminate (+/−) when fruit set ranged from 0.10 to 0.20; and compatible (+) when fruit set was higher than 0.20. Within each diallel, individuals were grouped according to shared incompatibility interactions to form incompatibility groups corresponding to shared *S* alleles (Brennan *et al.* 2002). *S* alleles were inferred to be interacting either dominantly or co-dominantly when individuals belonged to one or two incompatibility groups, respectively (Brennan *et al.* 2002).

The total number of *S* alleles present within each population (*N*) was estimated according to Brennan *et al.* (2002, 2013):

$$n=N{\left(1-\left(\frac{1}{N}\right)\right)}^{r},$$

where *n* is the number of *S* alleles identified in a sample and *r* the number of plants sampled. This maximum-likelihood estimator was modified from that developed for GSI systems (Paxman 1963) in order to make it suitable for SSI systems by assuming equally frequent *S* phenotypes (isoplethy) within panmictic populations rather than isoplethy of *S* allele genotypes to account for the presence of dominance interactions among *S* alleles. In addition, we calculated the repeatability index *R* (Stevens and Kay 1989) of our results in order to measure the thoroughness of the study:

$$R=1-\frac{n-2}{2r-2},$$

which ranges from 0 (as many different *S* alleles identified as *S* alleles sampled) to 1 (the minimum number of *S* alleles possible for a SSI system identified in the entire sample).

### Statistical analyses

We used SPSS software (SPSS for Windows, ver.15.0.1, 2006,Inc., Chicago) to perform statistical comparisons. After confirming that raw or transformed data were normally distributed using Shapiro–Wilk tests, we tested the relationship between both mean and log mean population ISI values and (i) log transformed effective population size (counts of flowering individuals) and (ii) density of plants (individuals/100 m^2^) by means of Pearson correlations using demographic data extracted from Silva *et al.* (2015*b*). We also performed Kruskal–Wallis and Mann–Whitney tests to compare (i) the fruit set obtained after each pollination treatment within every taxon; and (ii) the differences in fruit set between *FSP* and *ASP* treatments among taxa. We chose non-parametric tests for these comparisons to reflect the highly non-normal distributions of the fruit-set data with no p value correction for multiple testing due to the relatively small number of repeated tests (3 per treatment/taxon).

## Results

### Strength of SI

**Figure 2.**
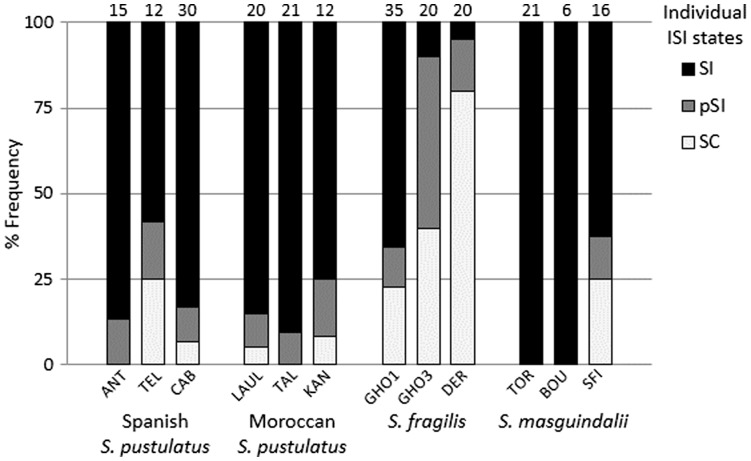
Strength and variation of the self-incompatibility across populations of the species of *Sonchus* section *Pustulati* assessed by the index of self-incompatibility (ISI; Lloyd 1965; Raduski *et al.* 2011). Individuals were classified according to the ISI states: self-compatible (SC, ISI < 0.2), partial self-compatible (pSI, 0.2 ≥ ISI < 0.8) and self-incompatible (SI, ISI ≥ 0.8). Numbers above bars are sampled individuals used to calculate frequencies of ISI states.

**Figure 3.**
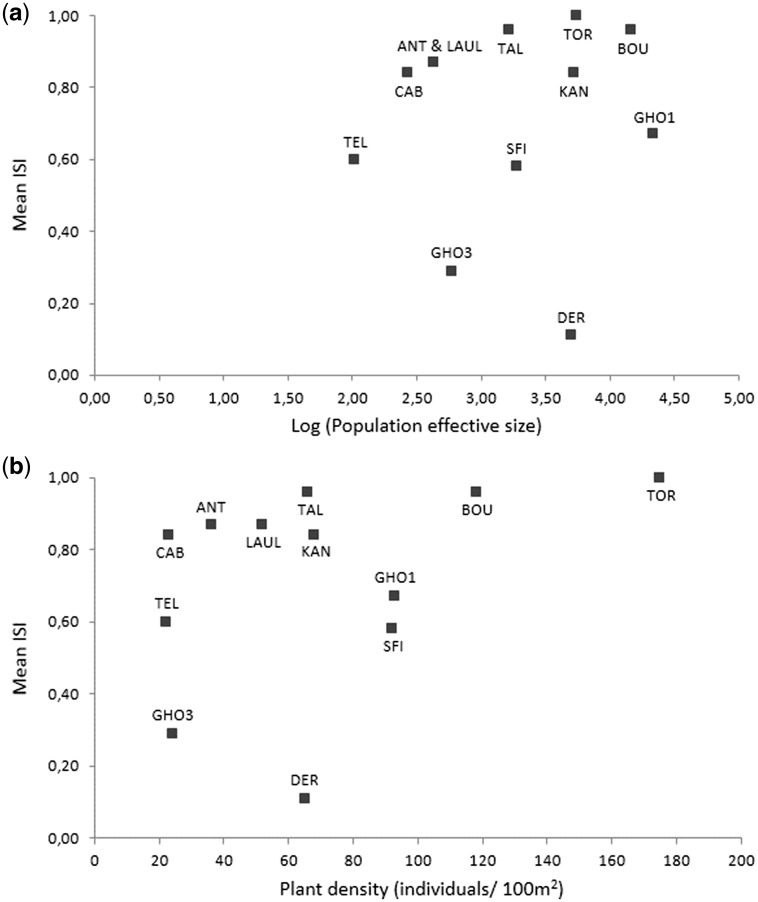
Relationships between the mean index of self-incompatibility (Mean ISI; Lloyd 1965; Raduski *et al.* 2011) across populations of the species of *Sonchus* section *Pustulati* and a) effective population size, and b) population plant density.

Strongly expressed SI was common in *S. masguindalii* and both the Moroccan and Spanish ranges of *S. pustulatus*, and these were classified as SI taxa (mean ISI = 0.84 ± 0.13, 0.90 ± 0.12 and 0.80 ± 0.11, respectively; Table 1). In contrast, *S. fragilis* showed a lower ISI and was classified as a pSI species (mean ISI = 0.42 ± 0.05; Table 1). At the population level, seven and four populations from the three species were classified as SI and pSI, respectively, and one population of *S. fragilis* (DER) was classified as SC (ISI = 0.11 ± 0.06; Table 1). At the intra-population level, the proportion of pSI and SC plants ranged from 0% to 50% and from 0% to 80%, respectively (Fig. 2). The mean ISI in populations was not related to the density of plants (*P* = 0.245) nor to the effective population size (*P* = 0.844; Fig. 3).

**Figure 4.**
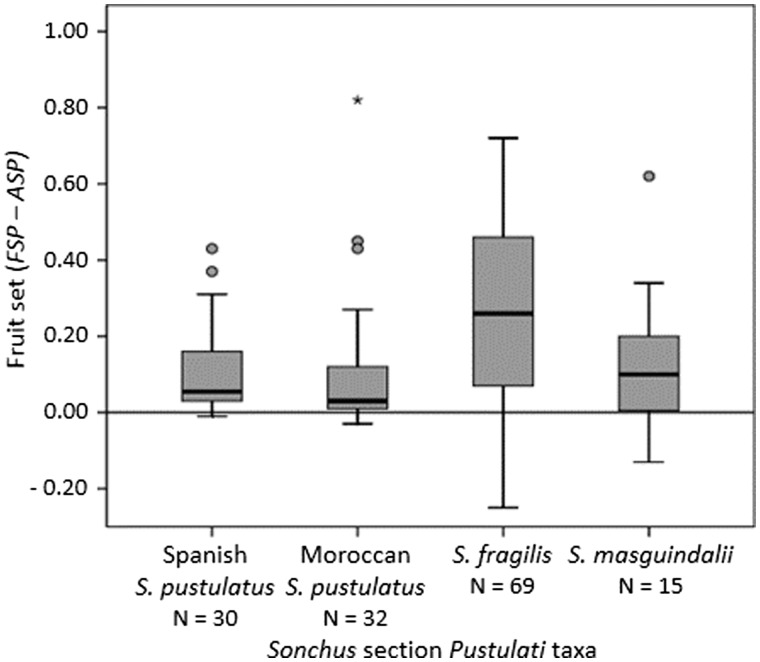
Variation of the individual difference on fruit set obtained after forced (*FSP*) and autonomous (*ASP*) self-pollinations across the *Sonchus* section *Pustulati* taxa. Small circles and asterisks indicate atypical and extreme values, respectively; i.e. outliers that are more than 1.5 and 3 box lengths from the upper hinge (75th percentile), respectively. Only plants that produced fruits after *ASP* and/or *FSP* pollinations were included (sample size = N).

The fruit set values for the *MCP* treatment were significantly higher than those of the *FSP* and *ASP* treatments in every taxon (Table 1; Kruskal–Wallis tests, *Χ^2^ *>96.22, DF = 2, *P* <0.001; Mann–Whitney U tests, *U* >0.001, DF = 1, *P* <0.001). Fruit set values after *FSP* were significantly higher than after *ASP* in each species (*U* >1673.00, DF = 1, *P* <0.020) except *S. masguindalii* (*U* = 1609.50, DF = 1, *P* = 0.117; [see **Supporting Information – Figure S2**]). In plants that produced fruits by selfing, after either *FSP* and/or *ASP* treatments, the difference between the fruit set obtained after these pollinations [i.e. Fruit set (*FSP* – *ASP*)] in each individual was 0.27 ± 0.03 in *S. fragilis*, 0.13 ± 0.05 in *S. masguindalii*, and 0.11 ± 0.02 and 0.10 ± 0.03 in the Spanish and Moroccan ranges of *S. pustulatus*, respectively (Fig. 4). This difference was significantly higher in *S. fragilis* than in the remaining taxa (*Χ^2 ^*=^* *^21.77, DF = 3, *P* <0.001; *U* >321.00, *P* <0.022). Surprisingly, some of these plants (10%), particularly of *S. fragilis* (6%), showed even a higher fructification after *ASP* than after *FSP*.

### *S* allele diversity estimation

**Figure 5.**
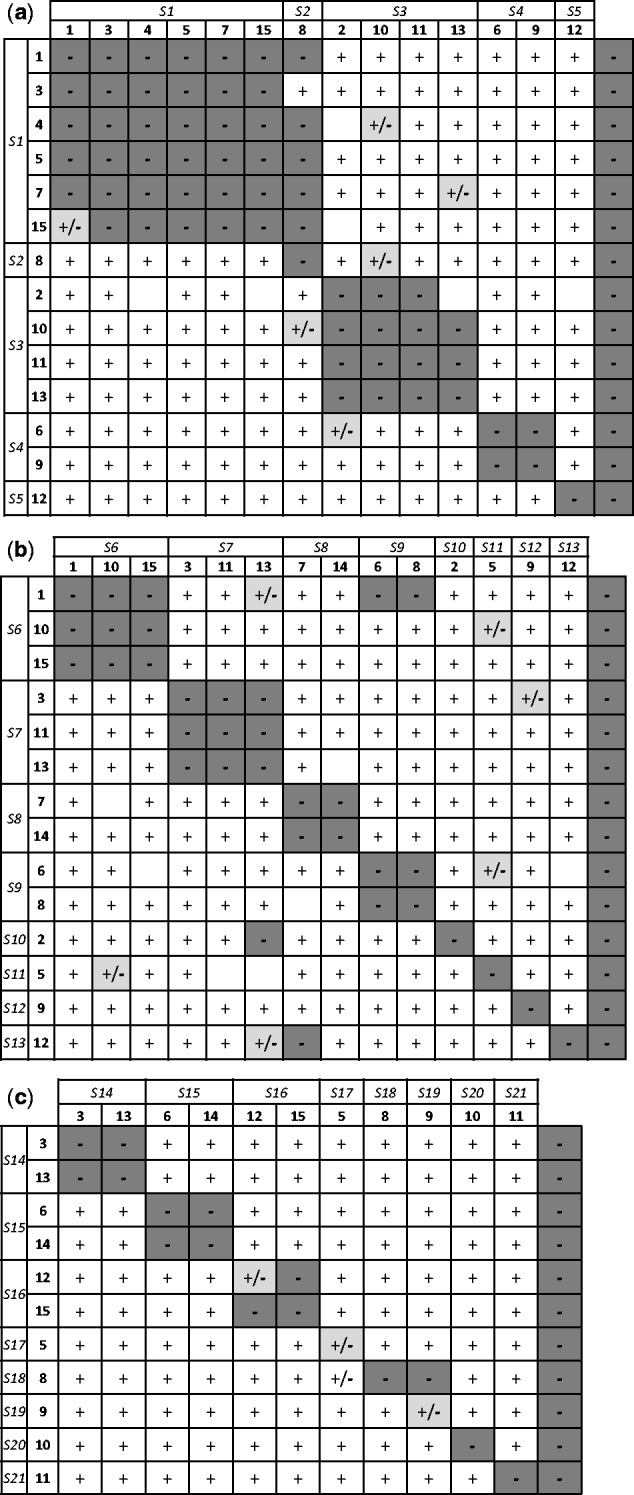
Analysed diallel of cross results between pairs of individuals from the Spanish population ANT of *Sonchus pustulatus* (a), the Moroccan population TAL of *S. pustulatus* (b) and the population GHO1 of *S. fragilis* (c). The first row and column refer to the dominantly or co-dominantly-expressed *S* alleles that have been identified. Codes of individuals acting as maternal or paternal are in the second column and second row, respectively. Symbols indicate compatibility reaction of crosses involving each pair of individuals according to the fruit set (see ‘Methods’ section): compatible (+ and white shading), incompatible (− and coloured or dark grey shading) and indeterminate (+/− and pale grey shading). No symbol indicates missing data. The last column refers to the compatibility reaction obtained after autonomous self-pollinations.

Our three complete diallels for populations ANT, TAL and GHO1 of Spanish and Moroccan ranges of *S. pustulatus* and *S. fragilis* allowed the identification of five, eight and eight incompatibility groups, respectively (Fig. 5a–c). These were interpreted as the numbers of different expressed *S* alleles in each population sample (Fig. 5a–c and [see **Supporting Information – Tables S1 and S2**]). It is worth noting that as the number of individuals used in the crosses was 14, 14 and 11, respectively, the number of potential alleles were 28, 28 and 22. We numbered the *S* alleles found from *S1* to *S21*, although individuals from different populations may share some of these alleles among them. From our diallel results, we interpreted *S* alleles to be expressed dominantly in those individuals belonging to a single incompatibility group. A SSI model of complete dominance among alleles satisfactorily explained the majority of the crossing results in each population (94.3–99.1% of results supported; [see **Supporting Information – Table S1**]). Furthermore, assuming co-dominance of alleles *S1* and *S2* in the pollen of plant 8 from ANT, and co-dominance of alleles *S6* and *S9* in the stigma of plant 1 from TAL [see **Supporting Information – Figure S3**], the SSI model explained a further 2.8 and 1.1% of the crossing results for each of these populations, respectively [see **Supporting Information – Table S1**]. Very few crossing anomalies, i.e. those not fitted into the SSI model, were detected across the diallels (0.9–4.6%; [see **Supporting Information – Table S1**]). The *S* allele number estimator of Brennan *et al.* (2002) predicted approximately 5, 11 and 15 *S* distinct alleles for the entire populations ANT, TAL and GHO1, respectively (Table 2). Repeatability values were 0.88, 0.77 and 0.70 for the three populations, respectively (Table 2), suggesting that sampling has been sufficient to capture most of the *S* allele diversity present in these populations.

**Table 2.** Analyses of the *S* allele diversity in the three populations sampled of *Sonchus pustulatus* and *S. fragilis*. *r*, number of sampled plants; *n*, number of *S* alleles identified in a sample, i.e. number of incompatibility groups identified in the sample (Hiscock et al. 2002); *N*, estimated number of *S* alleles present in the entire population; *R*, thoroughness of the study. ISI, index of self-incompatibility. N (pop.), effective population size; Mean fruit set, average fructification level in natural conditions; *H_E_*, average gene diversity based on AFLP data. * and ** according to Silva et al. (2015b, *a*, respectively).

Species	*S. pustulatus*						*S. fragilis*		
Distribution	SE Spain			N Morocco			N Morocco		
Population	ANT	TEL	CAB	TAL	LAUL	KAN	GHO1	GHO3	DER
*r*	14			14			11		
*n*	5			8			8		
*N*	5			11			15		
*R*	0.88			0.77			0.70		
N (pop.)*	426	104	269	1643	427	5198	21858	588	4963
Density (plants/100 m^2^)*	32	22	26	64	42	72	92	21	47
Mean fruit set*	0.66	0.46	0.58	0.78	0.77	0.76	0.63	0.76	0.85
H_E_**	0.088	0.101	0.093	0.146	0.138	0.155	0.146	–	–
Mean ISI	0.87			0.96			0.67		

The relatively high numbers of identified *S* alleles and small sample sizes preclude a formal statistical analysis of *S* allele spatial structure. Visual inspection suggested that the *S* alleles identified did not appear to show a strong spatial distribution structure within the populations apart from the population ANT, where the individuals of each of the incompatibility groups most represented in the diallels [those assigned with the *S1* and *S3* alleles; Fig. 5a and **Supporting Information – Table S2**] were mainly distributed in each one of the two clusters of individuals forming the population [see **Supporting Information – Figure S4**].

## Discussion

### Strength of expression of SI across *Sonchus* section *Pustulati*

The generally low fruit set observed after self-pollination (particularly autonomous pollination), relative to the large amounts of fruits for outcrossed treatments across individuals, indicate that *Sonchus pustulatus* and *S. masguindalii* have a relatively strong SI mechanism. This is not the case for *S. fragilis*, which according to our results has a weak SI system. Nevertheless, virtually all populations of *S. pustulatus* and *S. masguindalii* showed low proportions of SC and pSI individuals, which indicates that some flexibility of expression of SI is always present in the section. According to the ISI criteria used in the present study, the three *Sonchus* species of the section *Pustulati* would be therefore included in the long list of Asteraceae taxa that have been considered to bear a not always 100% efficient SSI system (i.e. SI-pSI species; e.g. Brauner and Gottlieb 1987; Reinartz and Les 1994; Young *et al.* 2000; Nielsen *et al.* 2003; Brennan *et al.* 2005; Ortiz *et al.* 2006; Lafuma and Maurice 2007; Scheffknecht *et al.* 2007; Ferrer *et al.* 2009).

Historical events involving major demographical constraints could have favoured the partial breakdown of SI (Mable and Adam 2007; Hoebe *et al.* 2009). A series of demographic, ecological and genetic features of these species described in Silva (2014) and Silva et al. (2015a, b) suggest an ancient origin and diversification, a relict condition and high ecological resilience despite a narrow ecological amplitude within a very restricted geographical distribution of the section *Pustulati*. The persistence of these populations could have been reinforced through the partial or complete breakdown of SI, decreasing therefore the *S* Allee threshold, that is, the number of *S* alleles under which a population shows a decline through scarcity of potential mates (Wagenius *et al.* 2007).

However, strongly expressed SSI was still the rule for the majority of individuals examined (Fig. 2 and Table 1), indicating that it is largely still an advantageous mating strategy. Selection to maintain strongly expressed SSI might be important for these long-lived perennial species where recessive deleterious mutations arising from mitotic mutation probably maintain high inbreeding depression (Morgan 2001). Studies of other cliff-dwelling species have found that they are frequently long-lived and persistent and have relatively stable population size (Larson *et al.* 2000; Picó and Riba 2002; García 2003; Lavergne *et al.* 2004; Thompson 2005; Silva *et al.* 2015*b*). This relative demographic stability might buffer against periods when the *S* Allee threshold of limited *S* allele numbers is passed and breakdown of SI would otherwise be selected for.

### Implications of highly pollinator-dependent self-fertilization for mixed mating systems

Differences in seed production between the *ASP* and *FSP* treatments were especially high in plants of *S. fragilis* (Fig. 4). Such a difference indicates that, despite of the capacity of self-fertilization, this species shows limited autonomous self-pollination. Therefore, SC and pSI plants likely need pollinators both to be either outcrossed or self-pollinated, and hence the mating system (from inbreeding to outcrossing) in the SC and pSI populations will primarily depend on the pollen load of pollinators and, secondly, on which type of pollen (self or non-self) has higher fertilization success [see **Supporting Information – Figure S5**]. Cross pollen appears to have a fertilization advantage in these mixed mating populations as evidenced by the higher values of fruit set detected after the crossed pollinations than in the forced self-pollinations. In the Asteraceae, SC taxa tend to constitute mixed mating systems rather than fully inbreeding populations (Ellstrand *et al.* 1978; Sun and Ganders 1988). For example, additional adaptations to promote cross-pollination such as attractive blooms (as for these *Sonchus* species) can lead to mixed mating outcomes for otherwise SC taxa. The use of molecular techniques would be necessary to determine the actual rates of self- and cross-fertilization in the SC and pSI populations of *Sonchus* section *Pustulati*. Another possible explanation for low *ASP* fruit set, also discussed later in terms of *S* allele diversity, is that the emergence of SC and pSI might be relatively recent in *S. fragilis* and complementary adaptations to increase autonomous pollination have not yet had time to evolve. SC alleles might nonetheless persist in these populations due to their automatic transmission advantage in any selfed progeny that are produced (Stone *et al.* 2014).

### *S* allele diversity

The results and interpretations from our three diallels support the presence of a sporophytic genetic control of self-incompatibility in the species of *Sonchus* section *Pustulati*. The high frequency of reciprocally compatible or incompatible inter-individual crossing results could be explained by *S* allele dominance interactions occurring both in pollen and stigma. Similarly, non-reciprocal compatibility or single incompatibility group crossing results could be explained by frequent *S* allele dominance interactions. The crossing results could thus be fitted to a sporophytic incompatibility model, and 5–8 incompatibility groups were identified in each of the three diallels supporting the multiallelic nature of the *S* locus (Brennan *et al.* 2002, 2006, 2013; Young and Pickup 2010).

It is of interest that the *S. fragilis* population was estimated to have the highest *S* allele number (15 versus 5–11 for *S. pustulatus*), despite showing higher levels of pSI. However, it is worth noting that other features of this population support the observation of higher *S* allele diversity. The GHO1 population is far larger than the S*. pustulatus* populations based on direct counts of flowering individuals and it has also maintained similar genetic diversity to these populations (Table 2). Therefore, the high *S* allele diversity suggest that the breakdown of obligate SI might be recent so that selfing has not gone on long enough to cause reductions in *S* allele number or heterozygosity.

The lower *S* allele diversity identified for the Spanish ANT population of *S. pustulatus* (5) compared to Moroccan populations (11–15) probably reflects greater long-term isolation on the Spanish side of the Alboran Sea compared to larger Moroccan populations of *S. pustulatus* that occur in relative proximity to *S. fragilis* and *S. masguindalii*. These related species might contribute to each other’s *S* allele diversity because negative frequency dependent selection favours the introduction of new *S* alleles between species even when migration and hybridization might be rare (Castric *et al.* 2008; Brennan *et al.* 2013). For example, Spanish and Moroccan individuals of *S. pustulatus* and *S. fragilis* seem to show high levels of interfertility (Silva, Mejías and Mendoza, unpubl. res.) but levels of interspecific sharing of *S* alleles have not yet been studied for these species.

The number of *S* alleles estimated by means of diallel crosses in three populations of two species from this group was relatively low (5–15) compared to the number of *S* alleles estimated in other species with SSI (range 2.1–54; mean 16.8; Busch *et al.* 2014). This number of *S* alleles was similar to another Asteraceae species so far studied, *Senecio squalidus* (7–11; Brennan *et al.* 2006), which also retained SSI despite a strong colonization bottleneck. A range of 4–22 *S* alleles were observed in samples of a second Asteraceae species, *Rutidosis leptorrhynchoides*, that were correlated with their population sizes spanning from 5 to 100 000 individuals (Young and Pickup 2010).

Similarly to *S. squalidus*, dominance interactions among *S* alleles were common in the three *Sonchus* populations sampled by means of diallels. Dominance interactions are thought to lead to a higher number of compatible crosses within a population than in the case of common co-dominant interactions among *S* alleles, so increase in dominance is predicted to be an evolutionary response to reduced *S* allele frequency (Byers and Meagher 1992; Brennan *et al.* 2003; Hiscock and Tabah 2003). The high levels of *S* allele dominance interactions observed for these species would further contribute to the resilience of their SI systems because more recessive *S* alleles are masked, thereby increasing population mate availability (Brennan *et al.* 2003). The evidence of stigma dominance interactions detected in the present study is consistent with the hypothesis that fecundity selection (i.e. reduced mate availability affecting female fitness) has been important in these populations ( Schoen and Busch 2009; Llaurens *et al.* 2009). It would be interesting to see how widely this SSI feature of frequent *S* allele dominance interactions might also apply to other narrow endemic taxa within the large Asteraceae family and other SSI families.

### Implications of SSI for the current negative demographic trends of Spanish *S.* *pustulatus*

The presence of pSI and SC plants and the dominance interactions among *S* alleles increasing the *S* Allee threshold may have been crucial for the relative demographically stability of the Spanish population ANT of *S. pustulatus* (Silva *et al.* 2015*b*), for which just five *S* alleles were estimated. However, in the case of the other Spanish populations CAB and TEL, which have been found to be in decline based on population viability analysis, these compensatory strategies do not now seem to be sufficient (see wild fruit set values Table 2, Silva *et al.* 2015*b*). The two populations (CAB and TEL) could have surpassed the *S* Allee threshold of adequate *S* allele numbers for reproductive success (Wagenius *et al.* 2007), although other factors such as low recruitment, increasing human disturbance or drought have also been implicated to be contributing to the population decline (Silva *et al.* 2015*b*). Any further slight decreases in the fruit set, due for instance to mate limitation, would further negatively affect the stability of these small populations.

All three Spanish populations of *S. pustulatus* showed signals of possible inbreeding depression in comparison with the Moroccan populations of the group for multiple demographical, reproductive and genetic features (Table 2; Silva 2014; Silva *et al.* 2015*a*, *b*). More empirical data about the level of inbreeding depression is required to better understand how SSI systems affect populations under threat of extinction as well as in the first stages of re-colonization after strong bottleneck events (Winn *et al.* 2011).

As habitat areas decrease and fragmentation increases, it becomes increasingly important to consider breeding systems when designing conservation plans (Wagenius *et al.* 2007). Here, we suggest that it would be beneficial to carry out a genetic rescue among the genetically impoverished Spanish populations of *S. pustulatus* (Silva *et al.* 2015*a*); at least from the most demographically stable and genetically isolated population ANT to the declining ones, TEL and CAB (Silva *et al.* 2015*a* , *b*). In a metapopulation where individual populations have lost *S* allele diversity, interpopulation crosses should more often alleviate mate limitation compared to crosses between plants from the same population (Busch and Schoen 2008; Young and Pickup 2010). As gene flow does not appear to now occur among the three Spanish populations (Silva *et al.* 2015*a*), it would therefore be useful for their conservation to perform interpopulation crosses in the field (Paschke *et al.* 2002; Willi and Fischer 2005; Pickup and Young 2008) or, due to low observed recruitment rates (Silva *et al.* 2015*b*), attempt the use of interpopulation cuttings transplants.

## Conclusions

This study highlights the value of performing detailed mating system studies in plant species of high conservation value, such as the rare endemic and relict species of *Sonchus* section *Pustulati* described here. This study adds to the evidence that outcrossing mating systems based on SSI are highly resilient even under long-term conditions of small, fragmented and isolated populations, possibly due to mating system flexibility with the presence of some selfing and the fact that high cross-compatibility is achieved for relatively modest dominantly expressed *S* allele polymorphism. We also find evidence for a mating system shift from SSI to facultative SC in some populations of *S. fragilis* suggesting the value of relict species as a system for studying mating system evolution. Finally, as additional anthropogenic and climate changes are placing additional pressure on vulnerable endemic and relict species, such as the Spanish populations of *S. pustulatus*, it will become increasingly important to take mating system factors into account as part of conservation efforts.

## Sources of Funding

This work was supported by a predoctoral grant to J.L.S. from the Spanish Ministry of Science and Innovation (BES–2007–17066) and a grant to J.A.M. from the Spanish Ministry of Science and Innovation (CGL2010–16512). Financial support for this research was provided by the Spanish Ministry of Science and Innovation (grants CGL2006–00817 and CGL2010-16512).

## Contributions by the Authors

J.A.M. conceived the project. J.L.S. performed the experiments and analysis. All authors, especially J.L.S., contributed to writing the article.

## Conflict of Interest Statement

None declared.

## Supplementary Material

Supplementary Data

## Supporting Information

The following additional information is available in the online version of this article—

**Figure S1.** Fertilized and unfertilized achenes.

**Figure S2.** Fruit (achene) set variation across the species of *Sonchus* section *Pustulati* according to the different types of pollinations applied over sampled individuals.

**Figure S3.** Different dominance and co-dominance relationships among *S* alleles in the stigmas and the pollen grains of some individuals inferred from results of diallel crosses.

**Figure S4.** Spatial distribution of sampled incompatibility groups in the populations.

**Figure S5.** Pollinator dependency for outcrossing levels in SC and pSI plants.

**Table S1.** Summary statistics for cross-classifications and the explanatory power of the sporophytic self-incompatibility model of near-complete *S* allele dominance interactions for the analysed diallels of cross results in the sampled populations.

**Table S2.** *S* allele designations for the individuals in the population samples.
