# Supplementary material for: Population genetics of self-incompatibility in a clade of relict cliff-dwelling plant species
Source: AoB Plants. 2016 Jul 11;8:plw029. doi: 10.1093/aobpla/plw029 (PMC4940477; doi:10.1093/aobpla/plw029)
Supplement: Supplementary Data [file supp_8_plw029_index.html]

Population genetics of self-incompatibility in a clade of relict cliff-dwelling plant species — Supplementary Data 

# Population genetics of self-incompatibility in a clade of relict cliff-dwelling plant species

## Supplementary Data

files

- Supplementary Data - zip file
