# Supplementary material for: Population genetics of self-incompatibility in a clade of relict cliff-dwelling plant species
Source: AoB Plants. 2016 Jul 11;8:plw029. doi: 10.1093/aobpla/plw029 (PMC4940477; doi:10.1093/aobpla/plw029)
Supplement: Supplementary Data [file supp_plw029_suppl_data_01.zip › aobplants-15293-s01.docx]

1) Topics selected from our subject list that best categorize your paper (please let us know if you think a topic needs to be added to our list).

Reproductive Biology; Genetics; Evolution; Conservation Biology.

2) An 80-100-word promotional statement that summarizes the results of your paper in a compelling way and is written in a relatively jargon-free style that is suitable for a general scientific audience.

This study highlights the value of performing detailed mating system studies in plant species of high conservation value, such as the rare and relict species of *Sonchus* section *Pustulati* described here. This study adds to the evidence that outcrossing mating systems based on SSI are highly resilient even under long-term conditions of small, fragmented, and isolated populations, possibly due to mating system flexibility with the presence of some selfing and the fact that high cross-compatibility is achieved for relatively modest dominantly expressed *S* allele polymorphism. We highlight the importance of taking mating system factors into account as part of conservation efforts.

3) A high-quality photograph or other image (preferably in colour) relevant to the manuscript (it may be one already included in the paper or a new image), with a brief explanatory caption that includes acknowledgement of the image source. Please aim for an image resolution of about 300 dpi at 10 x 10 cm and simply paste it into your cover letter. Permission from the copyright holder will be required for any images that are not original, including images from the Internet, unless they are considered to be in the public domain.

The figure has been attached as .pptx file.

Figure capture: Pollinator dependency for outcrossing levels in self-compatible (SC) and partially self-compatible (pSI) plants. As SC or pSI plants of *Sonchus* section *Pustulati* likely need of pollinators to be either outcrossed or self-pollinated, the mating system (from inbreeding to outcrossing) in the SC and pSI populations will primarily depend on the pollen load of pollinators and, secondly, on which degree of pollen (self or non-self) has higher fertilization success.

4) A list of up to 10 email addresses of colleagues to whom you would like us send a PDF link to your paper once it published fully-formatted.

[jesusval@us.es](mailto:jesusval@us.es),

[sonchus2009@gmail.com](mailto:sonchus2009@gmail.com),

[mariab@ipe.csic.es](mailto:mariab@ipe.csic.es),

[arroyo@us.es](mailto:arroyo@us.es),

[simon.hiscock@obg.ox.ac.uk](mailto:simon.hiscock@obg.ox.ac.uk),

[Barbara.Mable@glasgow.ac.uk](mailto:Barbara.Mable@glasgow.ac.uk),

[jwbusch@wsu.edu](mailto:jwbusch@wsu.edu),

[Pierre-olivier.cheptou@cefe.cnrs.fr](mailto:Pierre-olivier.cheptou@cefe.cnrs.fr),

Vincent.Castric[at]univ-lille1.fr,

[xavier.vekemans@univ-lille1.fr](mailto:xavier.vekemans@univ-lille1.fr)

5) The name of any Special Issue for which your paper is being prepared.

Not found in the list.
