# Supplementary material for: Population genetics of self-incompatibility in a clade of relict cliff-dwelling plant species
Source: AoB Plants. 2016 Jul 11;8:plw029. doi: 10.1093/aobpla/plw029 (PMC4940477; doi:10.1093/aobpla/plw029)
Supplement: Supplementary Data [file supp_plw029_suppl_data_01.zip › aobplants-15293-s02.docx]

**Table S3.** Summary statistics for cross-classifications and the explanatory power of the sporophytic self-incompatibility model of near-complete *S* allele dominance interactions for the analysed diallels of cross results in the three populations sampled of *Sonchus pustulatus* and *S. fragilis*.

| Species | *S. pustulatus* | | *S. fragilis* |
| --- | --- | --- | --- |
| Distribution | SE Spain | N Morocco | N Morocco |
| Population | ANT | TAL | GHO1 |
| Cross classifications (between pairwise of individuals) |  |  |  |
| Crossed classed as incompatible | 25.8% | 10.9% | 6.4% |
| Crossed classed as compatible | 67.6% | 82.4% | 93.6% |
| Undetermined crossed | 3.3% | 3.4% | 0.0% |
| Missing data | 3.3% | 3.3% | 0.0% |
| Explanatory power of the fitted SSI model |  |  |  |
| Crosses explained by designation of dominantly expressed *S* alleles | 94.3% | 94.3% | 99.1% |
| Crosses explained by designation of tissue-specific co-dominance  interactions between *S* alleles in plants 8 (ANT) and 1 (TAL) | 2.8% | 1.1% | 0% |
| Crosses not explained by *S* allele designations | 4.0% | 4.6% | 0.9% |
