# Supplementary material for: Population genetics of self-incompatibility in a clade of relict cliff-dwelling plant species
Source: AoB Plants. 2016 Jul 11;8:plw029. doi: 10.1093/aobpla/plw029 (PMC4940477; doi:10.1093/aobpla/plw029)
Supplement: Supplementary Data [file supp_plw029_suppl_data_01.zip › aobplants-15293-s03.docx]

**Table S4.** *S* allele designations for the *Sonchus pustulatus* and *S. fragilis* individuals in the three population samples, based on a sporophytic self-incompatible *S* allele dominance interpretation of the analysed diallels presented in Figure 7. Individuals with *S* alleles codominance interactions: (p) codominant in paternal pollen tissue but recessiveness to such allele in the in maternal stigma tissue; and (s), when the codominance interactions only occurs in the maternal stigma tissue.

| *S* allele designations | Plant | | | | | | |
| --- | --- | --- | --- | --- | --- | --- | --- |
|  | | | | |  |  |  |
| Population ANT of *S. pustulatus*, SE Spain | | | | |  |  |  |
| *S1* | 1 | 3 | 4 | 5 | 7 | 15 | 8(p) |
| *S2* | 8 |  |  |  |  |  |  |
| *S3* | 2 | 10 | 11 | 13 |  |  |  |
| *S4* | 6 | 9 |  |  |  |  |  |
| *S5* | 12 |  |  |  |  |  |  |
|  | | | | |  |  |  |
| Population TAL of *S. pustulatus*, N Morocco | | | | |  |  |  |
| *S6* | 1 | 10 | 15 |  |  |  |  |
| *S7* | 3 | 11 | 13 |  |  |  |  |
| *S8* | 7 | 14 |  |  |  |  |  |
| *S9* | 6 | 8 | 1(s) |  |  |  |  |
| *S10* | 2 |  |  |  |  |  |  |
| *S11* | 5 |  |  |  |  |  |  |
| *S12* | 9 |  |  |  |  |  |  |
| *S13* | 12 |  |  |  |  |  |  |
|  | | | | |  |  |  |
| Population GHO1 of *S. fragilis*, N Morocco | | | | |  |  |  |
| *S14* | 3 | 13 |  |  |  |  |  |
| *S15* | 6 | 14 |  |  |  |  |  |
| *S16* | 12 | 15 |  |  |  |  |  |
| *S17* | 5 |  |  |  |  |  |  |
| *S18* | 8 |  |  |  |  |  |  |
| *S19* | 9 |  |  |  |  |  |  |
| *S20* | 10 |  |  |  |  |  |  |
| *S21* | 11 |  |  |  |  |  |  |
