# Supplementary figures and images for: Population genetics of self-incompatibility in a clade of relict cliff-dwelling plant species
Source: AoB Plants. 2016 Jul 11;8:plw029. doi: 10.1093/aobpla/plw029 (PMC4940477; doi:10.1093/aobpla/plw029)

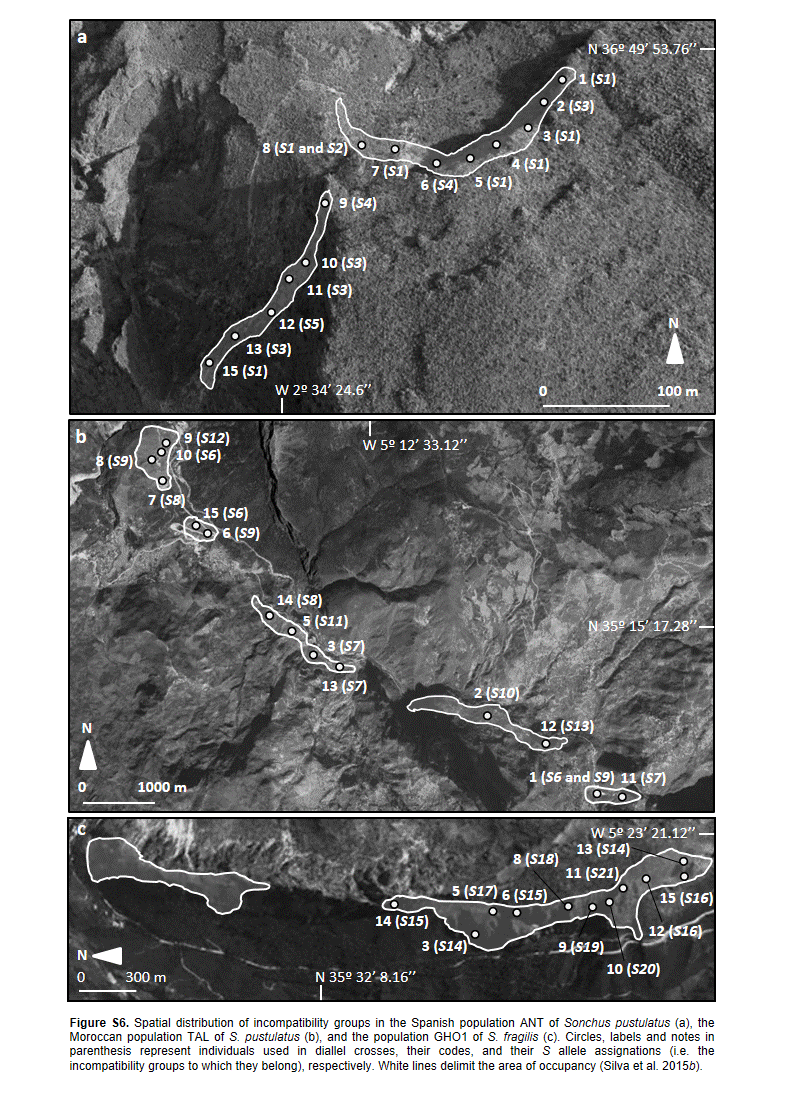

Supplement: Supplementary Data [file supp_plw029_suppl_data_01.zip › aobplants-15293-s07.gif]

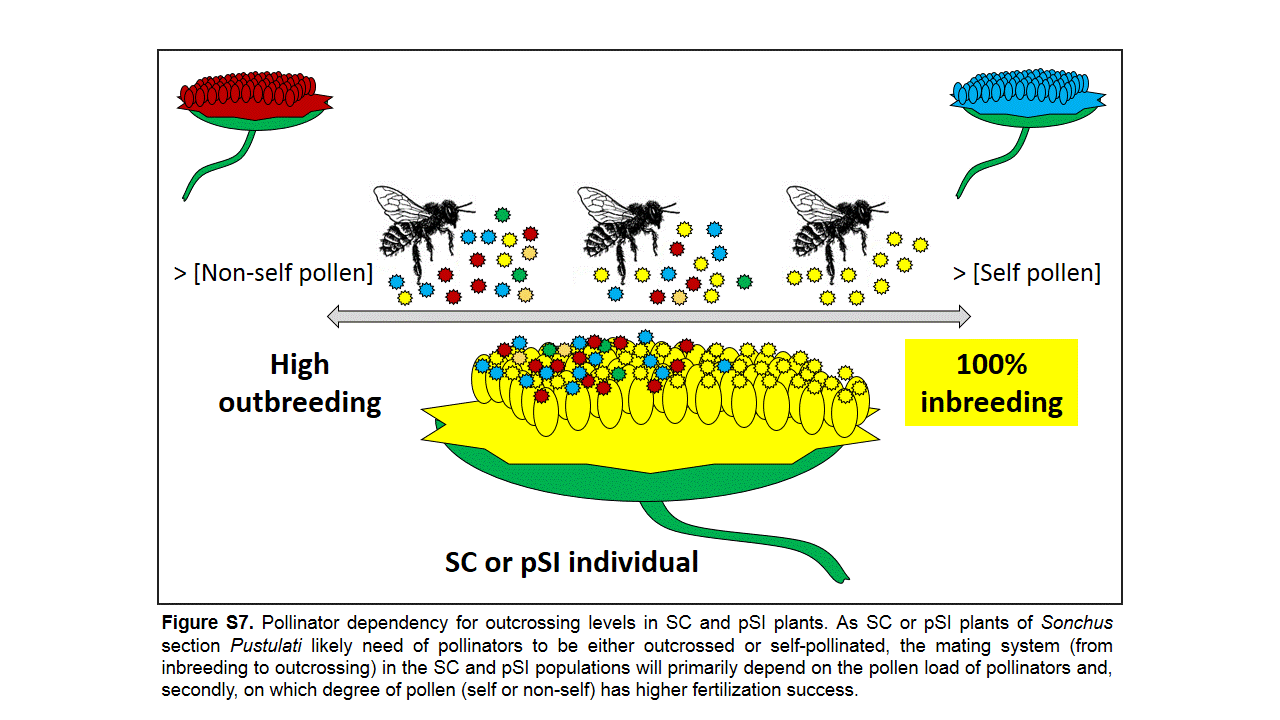

Supplement: Supplementary Data [file supp_plw029_suppl_data_01.zip › aobplants-15293-s08.gif]

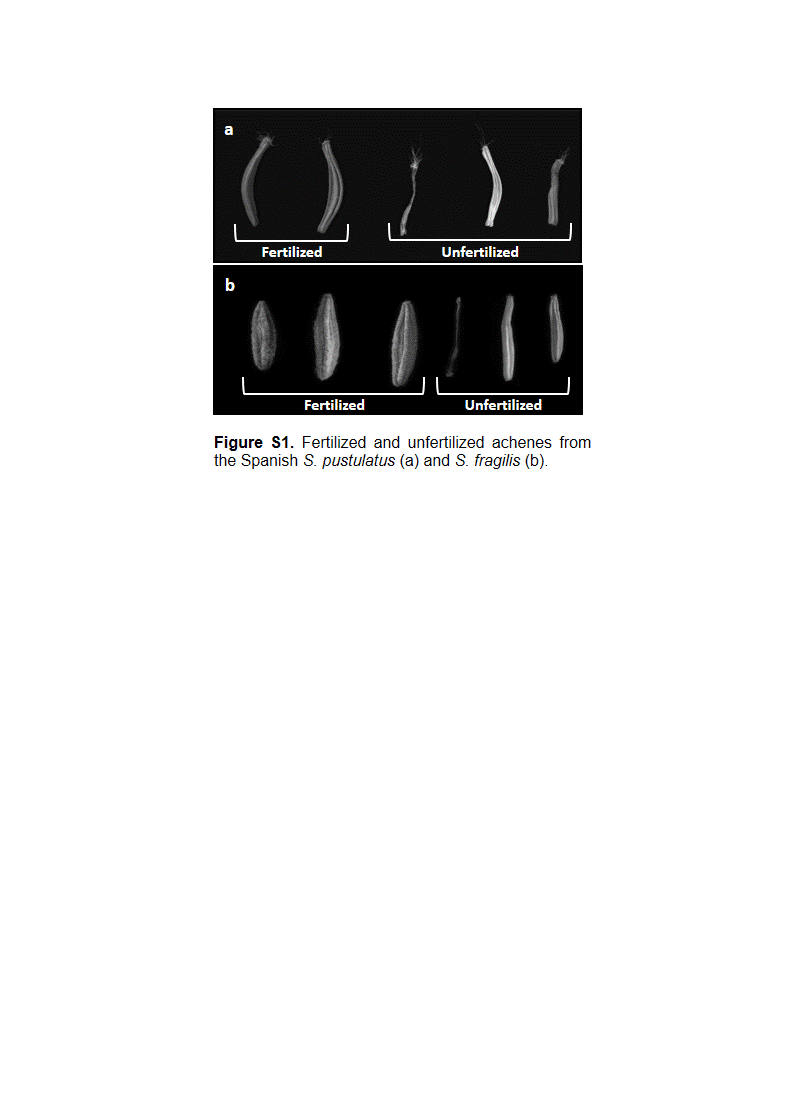

Supplement: Supplementary Data [file supp_plw029_suppl_data_01.zip › aobplants-15293-s04.gif]

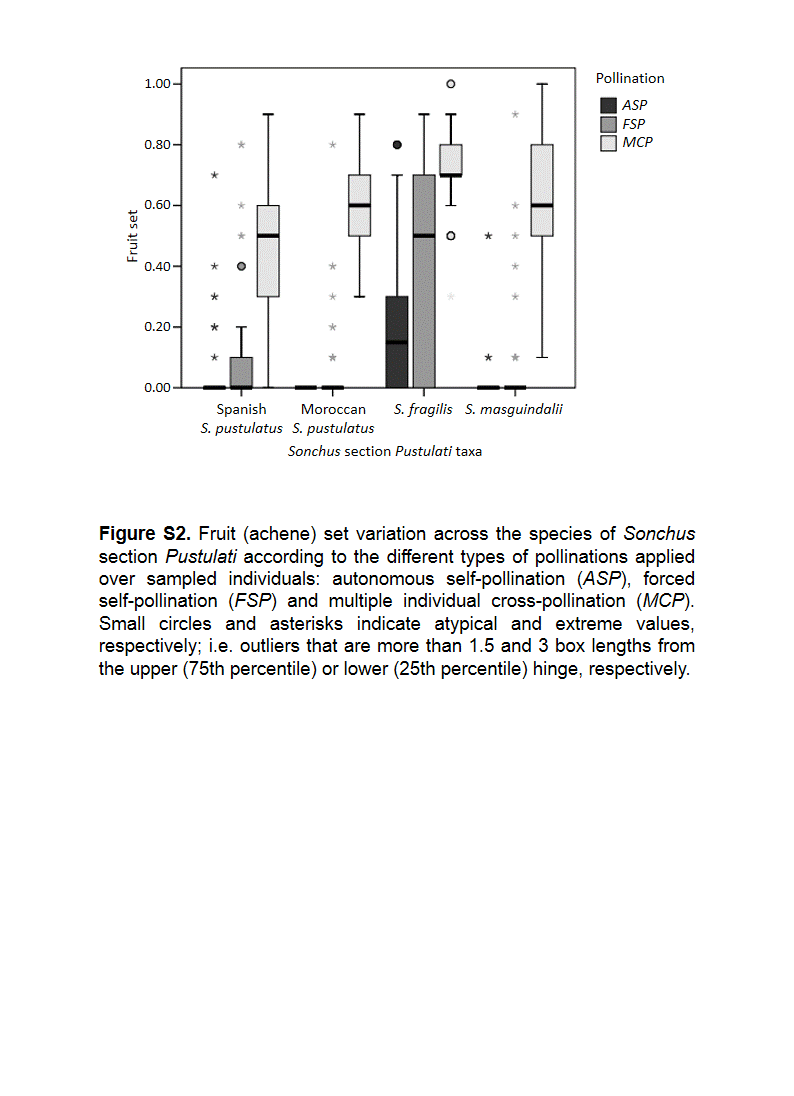

Supplement: Supplementary Data [file supp_plw029_suppl_data_01.zip › aobplants-15293-s05.gif]

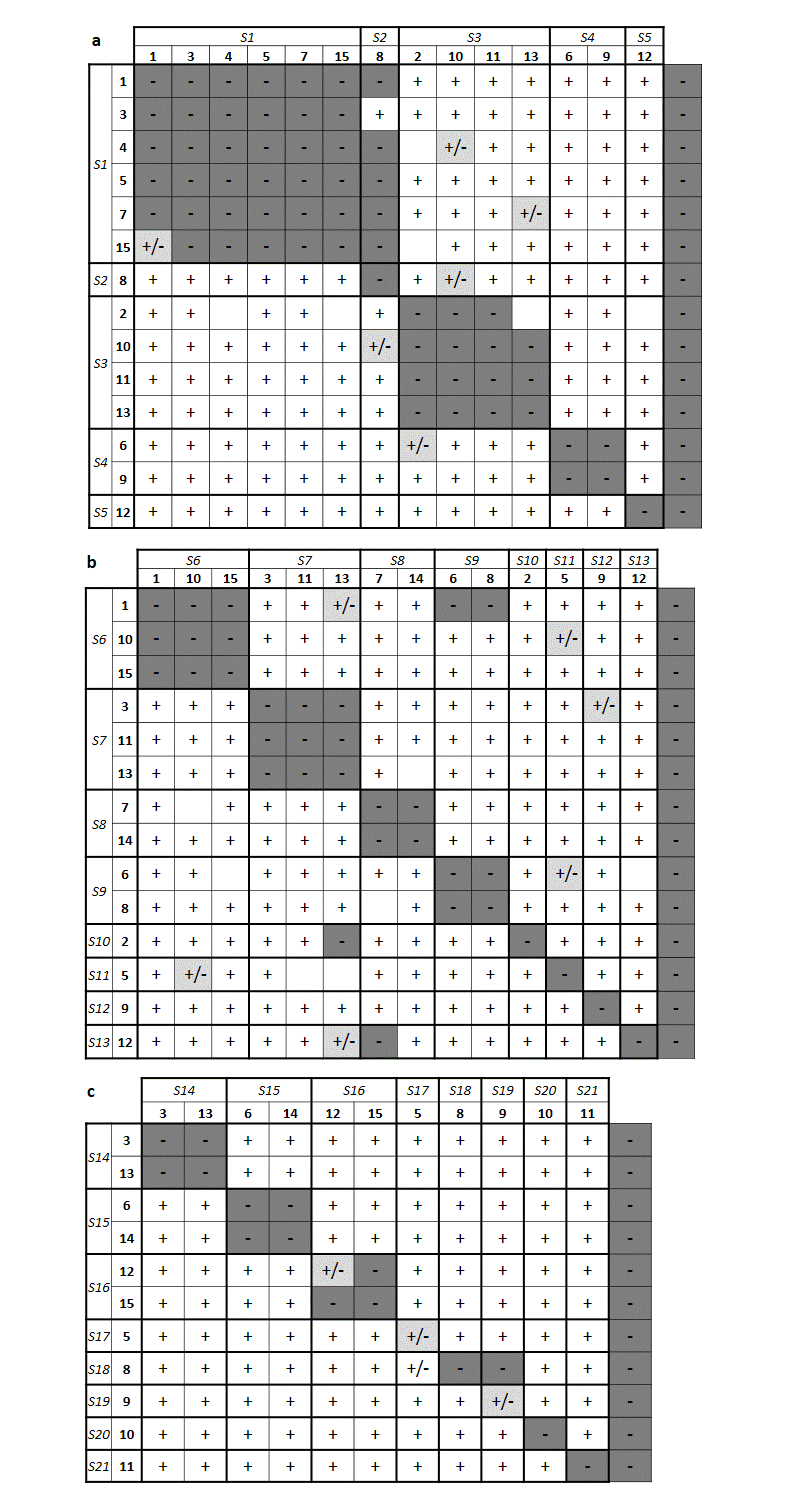

Supplement: Supplementary Data [file supp_plw029_suppl_data_01.zip › aobplants-15293-s06.gif]
